# Tracing the prescription journey: a qualitative evaluation of an interprofessional simulation-based learning activity

**DOI:** 10.1186/s41077-017-0047-0

**Published:** 2017-08-14

**Authors:** Caoimhe Cooke, Gerard J Gormley, Sharon Haughey, Johanne Barry

**Affiliations:** 1 Centre for Medical Education, Queen’s University Belfast, Belfast, Northern Ireland; 2 School of Pharmacy, Queen’s University Belfast, Belfast, Northern Ireland

**Keywords:** Interprofessional learning, Medical students, Pharmacy students, Simulation, Prescribing

## Abstract

**Background:**

In many countries across the world, the majority of prescribing occurs within the community setting. Close collaboration between general practitioners (GPs) and pharmacists is required to ensure effective therapeutic treatment of patients, whilst minimising prescribing and dispensing errors. Despite the need to work collaboratively, medical and pharmacy training is often unilateral. Interprofessional education (IPE) and simulation-based education (SBE) are teaching approaches widely used by healthcare professionals to foster collaborative practice. At Queen’s University Belfast (QUB), an innovative IPE activity was developed for medical and pharmacy undergraduate students that aimed to develop a greater understanding of their roles and duties in community prescribing and dispensing. This study set out to evaluate the impact of such a SBE activity on students’ attitudes towards collaborative practice in prescribing and dispensing medication in the community.

**Methods:**

Interprofessional groups of year 3 pharmacy (*n* = 10) and year 4 medical (*n* = 9) students took part in a SBE activity. This focused on the IPE team clinically assessing, diagnosing, writing prescriptions, dispensing medication(s) and counselling a simulated patient (in a simulated practice and pharmacy setting). Using a questioning guide, four focus groups of medical and pharmacy students were used to evaluate their attitudes towards the simulated IPE activity. Interviews were audio-recorded, transcribed and analysed iteratively using thematic analysis.

**Results:**

Four main themes emerged from the analysis: (1) IPE simulation activity: creating a broader learning experience; (2) patient-centred practice: a shared understanding; (3) professional skills: explored and shared; and (4) professional roles: a journey of discovery, respect and stereotypes.

**Conclusions:**

Students broadened their knowledge of each other’s expertise in skills and clinical roles whilst working together. Furthermore, students valued the opportunity to strengthen cooperation with their future colleagues with the shared goal of improving patient-centred care.

## Background

Prescribing and dispensing of medications are complex, yet routine clinical tasks. There are many steps involved in both activities including clinical assessment, establishing a diagnosis, devising a management plan, drug selection, labelling and patient education. The supply of prescribed medicines to patients in the community is a collaborative process shared among many healthcare professions, most commonly general practitioners (GPs) and pharmacists. Despite the routine nature of this task and a drive for greater community-based healthcare, there is an under-representation of interprofessional learning among undergraduate medical and pharmacy students highlighted by Dornan et al. [1].

Interprofessional education (IPE), a teaching method endorsed by the World Health Organisation, aims to develop the skills and knowledge required to be a collaborative health worker. Successful IPE can afford students a deeper understanding of the roles of their co-professionals, in result optimising the skills of their health teams and improving health outcomes [2]. Simulation-based education (SBE) is a widely used teaching method that provides learners with an opportunity to rehearse and advance their skills before transferring them to clinical practice, in a safe environment without compromising patient safety [3]. SBE can be used alongside, and as a complement to, more traditional education methods. Historically, SBE has an emphasis on advancing acute care skills such as cardiac arrest team responses [4] and emergency airway management [5]. However, SBE is offering new types of learning experiences as this pedagogic paradigm develops.

At Queen’s University Belfast (QUB), we have developed an innovative IPE activity offered to medical and pharmacy undergraduate students. This learning activity, using simulation techniques including a simulated dispensing pharmacy, aims to offer medical and pharmacy students a deeper understanding of each other’s role in prescribing, dispensing and guiding patient education within the community.

### Aim of project

The primary aim of this project was to qualitatively evaluate the impact of this SBE activity on students’ attitudes towards IPE when prescribing. Secondly, we aimed to ascertain student perceptions on the value of this prescribing- and dispensing-focused SBE activity and how well the SBE activity supported their core teaching and professional development.

## Method

### Setting and context

At QUB, the medical degree programme follows a 5-year undergraduate curricular model and the pharmacy degree programme follows a 4-year integrated, spiral curriculum model. As part of their core curriculum, the teaching took place in the simulated pharmacy practice unit in the School of Pharmacy (SoP). This unit includes tutorial rooms and a simulated community pharmacy.

### Description of interprofessional SBE innovation

**Fig. 1:**
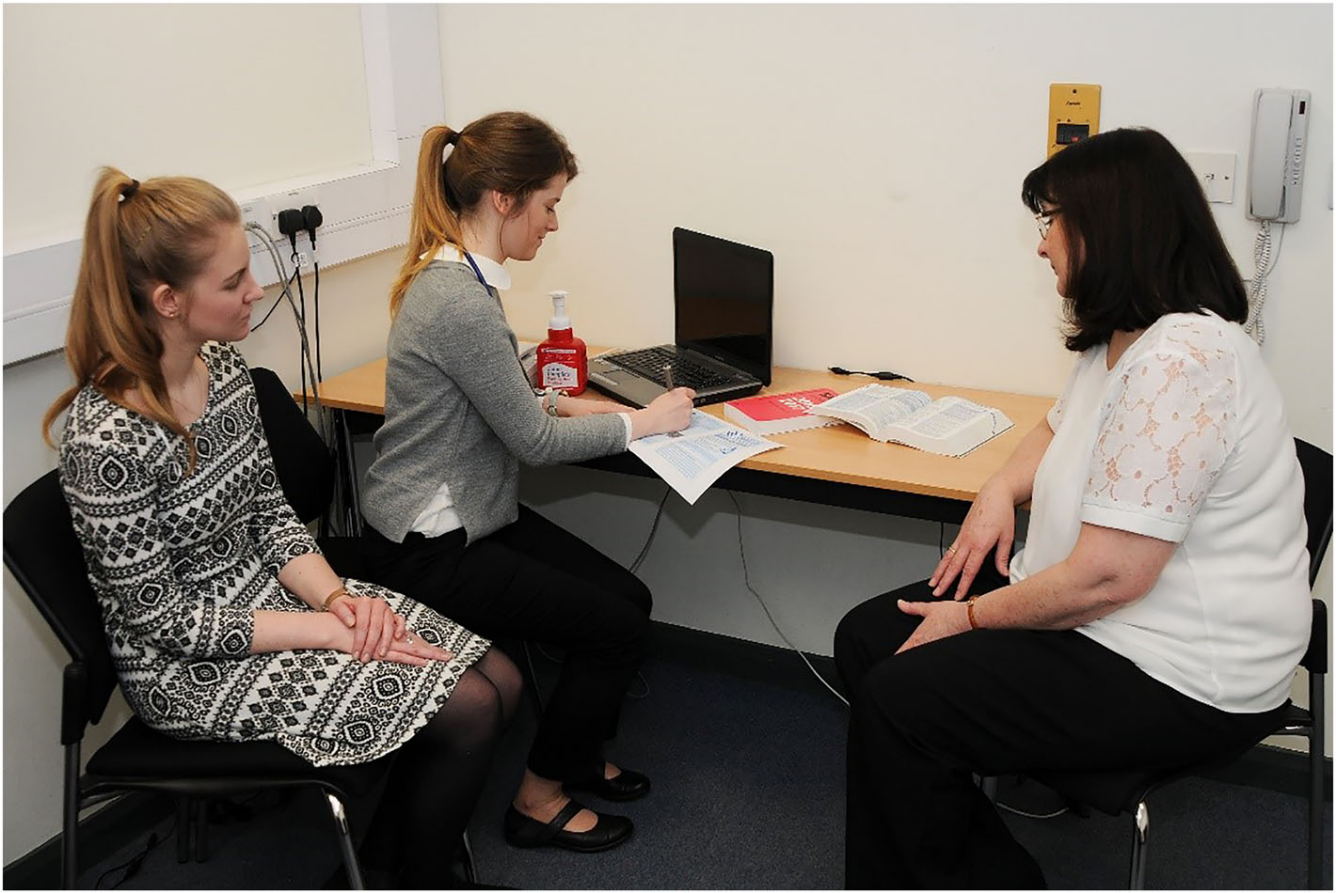
Interprofessional group of medical and pharmacy students clinically assessing a simulated patient

Groups of medical and pharmacy students are initially briefed about the simulation activity. Learning objectives were provided to encourage a shared understanding of the exercise. Then, in small mixed-disciplinary groups, they were asked to consult with a ‘patient’ (i.e. a simulated patient who had been briefed about their role) in a simulated GP office. The simulated patients described common general practice presentations (e.g. back pain, sore throat, primary cardiovascular risk assessment and request for emergency contraception) (see Fig. 1)*.* During this ‘consultation’, students were provided with a medical chart relating to the patient, access to a drug formulary (i.e. the British National Formulary) and other diagnostic equipment. Typically, during this ‘consultation’, the medical student would lead, with the pharmacy student actively observing.

**Fig. 2:**
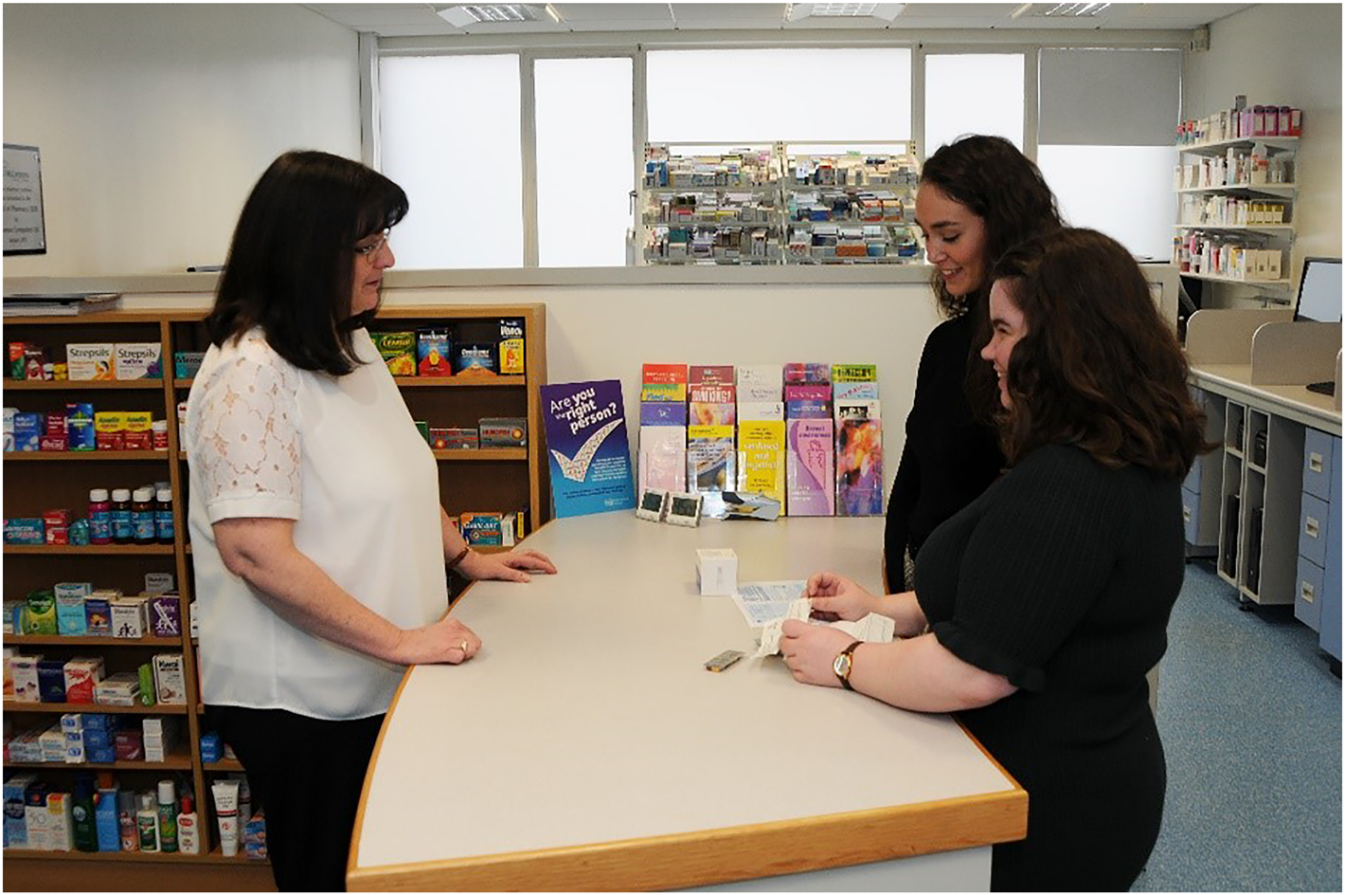
Interprofessional group of medical and pharmacy students preparing and dispensing ‘medication’ to a simulated patient

Following their assessment, the simulation was paused. The student small groups were tasked to consider a working diagnosis for the patient. They then collaborated on writing a mock drug prescription and detailing a management plan (both pharmacological and non-pharmacological aspects). Following this, they engaged with the simulated patient again to explain their working diagnosis and negotiate their proposed management plan. After this was complete, the simulated patient attended the ‘simulated pharmacy’ for the dispensing of their medication (see Fig. 2).

As in most simulated pharmacies, they contain a stock of common medications, computer software normally used in dispensing, set in the physical layout of a typical pharmacy (i.e. dispensing area, patient consultation counter)*.* During this part of the exercise, the pharmacy students usually took the lead and explained their actions to the medical students. The patients’ medication prescription was processed and dispensed. They also received counselling about their medication and follow-up arrangements.

Finally, the small groups were debriefed by faculty staff from the medical and pharmacy schools and the simulated patients. In these debrief sessions, feedback was actively encouraged between the two different groups of healthcare students in an attempt to reinforce interprofessional values and understanding. Whilst the simulation activity was not video-recorded, the faculty used their observations and field notes to guide the debrief session. The session concluded with a review of the evidence base on how best to manage their patients ‘conditions’.

### Recruitment and sampling

Using a convenience sampling method, we recruited year 4 medical students and year 3 pharmacy students to participate in focus groups following their participation in the IPE simulation-based teaching activity. Two mixed focus groups (i.e. a mixture of 3–6 medical and pharmacy students in total in each group) and two uniprofessional (i.e. separately 3–6 medical or pharmacy students in each group) were convened in order to encourage active discussion across and within professions. Informed written consent was obtained from participants. Ethical approval was obtained from the Schools Research Ethics Committee in advance of the study (Ref: 011PMY2016).

### Data capture

Interviews were guided by a question guide which was based on a review of the literature. The focus groups were exploratory, deriving from what was shared to remain rooted in their experiences. All focus groups were digitally recorded and transcribed verbatim. Citations, below, are coded: female or male participant, pharmacy or medical participant (pharm/med) focus group number (FG) (e.g. female, pharm FG2).

### Analysis

Analysis was led by a researcher (CC) who was not directly involved in with the creation (or delivery) of the simulation teaching innovation. Transcripts were reviewed independently by all members of the research team (i.e. including pharmacy and medical academics). After the first independent readings, the researchers agreed on dimensions for further focus, concentrating on participant’s attitudes towards the IPE SBE activity, the value they placed on the process and their professional development. The inductive analysis process began with the research team meeting regularly to discuss and review the emergent themes uncovered based on importance and relevance to the evaluatory aim. The researcher’s methodical consultation of the transcripts ensured the themes were rooted within the data. Team reflective checks were included to minimise any distortion of the analysis by researchers’ preconceptions, assumptions and opinions. Finally, the research team reached consensus on the main themes of the data.

## Findings

In total, 19 students (9 medical and 10 pharmacy) formed four focus groups that generated 92 min of interview data. Detailed analysis of the data yielded four main themes of participant’s experiences of the interprofessional simulation-based prescribing and dispensing activity: (1) IPE simulation activity: creating a broader learning experience; (2) patient-centred practice: a shared understanding; (3) professional skills: explored and shared; and (4) professional roles: a journey of discovery, respect and stereotypes.

### IPE simulation activity: creating a broader learning experience

During their prior training, the participants had only a small number of interprofessional learning experiences, often being more theoretically orientated such as class-based tutorials. Together, pharmacy and medical students discovered that the simulated environment offered them an opportunity to apply their theory knowledge into ‘practice’ collectively and safely. Both professions expressed that this method of teaching was perceived to be more beneficial to them, particularly the simulated environment contributing to their understanding and learning of the social and interpersonal dimensions of prescribing and dispensing.

You learn better practically, a lot better than you do being in a lecture. You’re always going to take more away from these sort of sessions than watching an hour’s lecture. (Male, Med FG2)

…obviously in first and second year it was more sitting in a room and going over calculations or something, whereas this is a lot more interactive. I really enjoyed today, it was really good to see it. (Female, Pharm FG2)

The teaching of prescribing can often have a dominant theoretical perspective. However, with the use of a simulated patient further enhanced the realism of this simulation. The participants particularly highlighted that the simulated patients provided them with a more realistic view of the problems that can arise in actual practice.

So they had a sore throat, they didn’t know if they could take tablets, so there’s a bit more counselling and thinking on your feet there ….. it’s quite realistic; patients will have problems in practice and I think this was reflected in the scenario…. (Male, Pharm FG1)

In addition to the participants developing their consulting, prescribing and dispensing skills together, the activity also allowed them to foster their mentorship abilities. Medical students felt that they benefited from the pharmacy students guiding the prescription writing and dispensing sections, as many of them had little understanding of these processes. Conversely, medical students led in history taking enabling the pharmacy students to ‘tailor their consultations’ with the simulated patients after dispensing their medications. As with many other simulation-based teaching methods, the participants appreciated the relative safety and supportive environment of the simulation.

We are going to be writing scripts in a few years’ time so I kind of liked it that they were there and we able to bounce of each other and tell them about what questions we would ask, how we counsel them and they reiterated that and said they liked being able to see what we were telling the patient… (Male, Med FG3)

### Patient-centred practice: a shared understanding

The use of simulated patients (SPs) in the simulation very much triggered a critical reflection among students about patient-centred practice. This was particularly enhanced when each profession of students was able to consider how other professions demonstrated patient-centeredness. As often with prescribing, there can be dominance with intellectual learning, with risk of rendering the person (patient) invisible in the process. However, having a ‘human face (i.e. the SP) reinforced the ‘person behind the prescription’—reminding students that the skills they were refining—served to benefit the care of their future patients.

For many medical students, this was their first opportunity to appreciate the dispensing process, and so, admittedly, the continuous focus on patient-centeredness and safety came as a pleasant surprise to them. Steps taken by the pharmacy students, for example, the appropriate use of capital letters on medicine labels and counselling the patient about their medication, impressed the medical students who had not fully considered this aspect of the patient journey in prescribing

it’s opened my eyes to how patient-centred the dispensing and counselling really is. (Female, Med FG2)

Often in the community, patient consultation, prescription writing and dispensing processes are disconnected in students learning. Witnessing each component stage of the patient journey, from GP consultation to receiving medications, gave pharmacy students a critical insight into the patient experience that many had not encountered before. They expressed that speaking with the simulated patients humanised the ‘prescriptions’ written on paper and trained them to dispense and consult in a more empathetic way.

…to be empathetic, you are not just treating a piece of paper you are treating a patient there is a person lies at the end of it (Male, Pharm FG4)

### Professional skills: explored and shared

The session highlighted two main skill sets among both professions as areas of development and expertise: prescription writing and consulting with patients. Medical students found prescription writing by hand particularly challenging, having more experience with hospital drug charts and computer-generated prescriptions on clinical placements. Pharmacy students, who had cultivated this skill over their years of study, ensured the format, dosages and instructions of the medical student’s ‘prescriptions’ were written legally and reflected good prescribing practice.

…it was helpful to see the pharmacists being able to show us how they actually make out a prescription, knowing if it’s a capsule or a tablet. As a medical student, you probably wouldn’t even be thinking of that (Female, Med FG1)

Pharmacy students praised the holistic approach medical students applied to their consultations, examining many aspects of the simulated patients’ health including their acute presentation. However, some pharmacy students commented that medical students could often ‘bombard’ simulated patients with questions which seemed irrelevant. This served as a useful advice for future history taking by the medical students.

Something I thought was good was with our patient they weren’t just asking about drug therapy. They gave other options. Our medical student suggested yoga and that sort of thing, lifestyle things that could help, something other than drugs. (Female, Pharm FG2)

Medical students admitted to being unaware that pharmacy students are trained in providing patient education on drug interactions, side effects and dosage regimens. This made medical students feel supported that drug information relayed to patients during GP consultation would be reiterated by their pharmacy colleagues in the community. Participants from both professions noted that the IPE activity strengthened the trust and reliance they had in each other’s knowledge and skills.

I think knowing a pharmacist is checking your prescriptions, they’re the ones that have more knowledge in that area, that you’d go ‘I would like their expertise on it’ over yours. (Female, Med FG1)

### Professional roles: a journey of discovery, respect and stereotypes

Participants began to delve into a deeper understanding of the roles each play within their professions. Many differences in attitudes, between the two professions, towards practice were found. Notably, medical students were surprised at pharmacy student’s strict adherence to stipulated guidelines. This appeared to create a ‘natural tension’ between them, namely the use of clinical guidelines is promoted in pharmacy education at an early stage in the interest of patient safety. In contrast to this, medical students are encouraged to develop a more flexible approach to prescribing: ‘guidelines not tramlines’.

This fundamental variation in practice can be misinterpreted by pharmacy and medical practitioners giving rise to professional stereotypes. From the discussions, pharmacy students commented that medical students can often come across as arrogant or ‘cavalier’ in their approach to prescribing. Medical students supported this misconception but added that this activity allowed them to observe the dispensing process and understand how professional conflicts arise.

…in that you do learn to play to each other’s strengths and work as a team and there’s no point in that sort of cavalier approach of ‘I know best’, because it’s rarely what’s best for the patient. (Male, Med FG1)

It was unearthed that pharmacy students and pharmacists can be misjudged as the ‘nag’ character. It was implied that students assumed communications between pharmacists and GPs centred solely on following up the use of medications outside of recommendations, a perceived annoyance for medical students.

I think doctors often see pharmacists as a bit of a nag, but for us the legal requirements are so important on our side so we have to get things sorted out. But for doctors, they see that as annoying, just because it does take time for them. (Female, Pharm FG1)

Students valued the opportunity the simulated activity gave them to view these conflicting ideas in a more empathetic way. Pharmacy students gained an appreciation of how GPs may be trying medications outside of the recommended guidelines and medical students the importance of following guidelines for the benefit of patient safety, which will hopefully benefit both groups in their future practice.

The idea of the ‘production line’ dispensing process was discussed by pharmacy and medical students. The role of a pharmacist in providing optimal medications can often be underestimated [6], patients and doctors alike may presume dispensing medications to be a pharmacist’s exclusive occupation. This activity was the first occasion many of the medical students had to appreciate the multiple roles of a pharmacist, including dispensing, independent prescribing, referral, consultation and examination.

…I think it’s important that the doctors understand that we’re not just a production line, pill counters or whatever, but we do have that clinical knowledge and know our boundaries, when to pass it on for further investigation or whatever. (Male, Pharm FG1)

## Discussion

The simulation-based interprofessional activity provided pharmacy and medical students a beneficial opportunity to work together in promoting their professional development and critically reflecting on how best to work collaboratively in the assessment and treatment of patients. Focus group discussions following the activity revealed the attitudes students had towards the session and their professions. Students found that the activity helped them to address the strengths and limitations of their own prescription writing and consultation skills as well as broadening their initial knowledge of each other’s professional roles. As well as this, students expressed the value they saw in the activity in improving their future practice among professions and their future interactions with patients.

On analysis of the data, the first theme focused on the students’ views of the IPE activity itself. The students from both professions collaborated and mentored each other in a new learning experience which gave them better insight into their complex skills and roles. Pharmacy students expressed that the presence of simulated patients gave the scenarios an empathetic aspect and highlighted the importance of patient-centred care. Continuing from this, theme 2 emphasised the advantage of witnessing the entire patient journey from consultation to receiving medications. Students began to understand how the collaboration between them during the activity could greatly benefit the care of their patients in the future. Theme 3 looks at how, together, pharmacy and medical students explored the strengths and limitations of their own professional skills and shared their knowledge to further each other’s learning. Pharmacy students were surprised at medical student’s prescription writing skills, learning that much of their previous teaching had been computer-generated or ward-based. Medical students were impressed by pharmacy student’s consultation skills, many not knowing this was a skill they have nurtured throughout their pharmacy teaching. The final theme looks deeper at professional hierarchies and stereotypes between pharmacy and medical students and their roles in clinical practice, for example, medical students having an intimidating ‘cavalier’ air, pharmacist’s adherence to drug guidelines as ‘nagging’ and the ‘production line’ idea of dispensing. Students felt that cooperation in this activity would assist in breaking down these stereotypes and aid future practice together.

### Comparison with previous research

The previous IPE- and SBE-based research returned similar findings in relation to healthcare students and professional’s attitudes towards proposed interventions. SBE is common in teaching acute care techniques; however, there has been recent research into its efficacy in primary care. One particular study following a similar simulated patient design stated that ‘creating a service plan for the case required communication and collaboration between the disciplines and promoted a better understanding of the roles played by each group on the team’ [7]. Comparably, undergraduate views in an IPE systematic review commented that ‘real life scenarios on clinical wards encouraged team approach and collaboration through real experience’ [8]. This is echoed by pharmacy and medical student opinions towards this session; the simulation aspect promoted efficient cooperation between the students with a more realistic context. Previous research has also proposed the idea that IPE is well facilitated by case-based learning [9] which is supported by the responses of the students in our analysis. Although the structured activity gave students the opportunity to learn from each other, interestingly, pharmacy and medical students expressed simply being comfortable with each other and talking with their interprofessional peers further improved co-professional relationships. Some have noted the idea that non-classroom-based, informal networking parallel to IPE interventions should not be over-looked in the development of IPE curricula [8].

Stereotypes and hierarchies among professions are well documented within IPE research, Honan et al. even questioned whether pre-formed misconceptions among students could affect future attempts of collaborative practice [10]. Interprofessional education is grounded in contact theory: the idea that bringing members of different groups together should reduce prejudices; however, recent research has suggested that forcing multi-professional groups into interactions can confirm stereotypes if equal status among participants is not ensured [11].

The sense of mentorship that was provided by each profession following this activity contributed to the students regarding each other with equal status. Pharmacy students commented that medical students can often seem intimidating in their knowledge at times, a notion which has been recorded before. In a 2014 study of pharmacy and medical students partaking in an IPE intervention, doctors were said to be perceived as ‘intimidating’ [12], which was echoed in a 2015 longitudinal IPE study, acknowledging that after spending time with the medical students, this was a misjudgement [13]. Medical students have also been reported to respond with a lower willingness to collaborate than pharmacy students [14] and unopposed to the idea that the role of the pharmacist is nothing more than supportive to the work of a doctor. These impressions were not articulated among the medical students in this study; in fact, the importance of working together was repeatedly stated and that the activity helped medical students rely and trust in the expertise of their pharmacy colleagues.

Stereotypes of pharmacy students’ over-orientation on faultfinding and adherence to drug guidelines is a reiteration of previous research [13]. However, being afforded the experience of following the dispensing process, medical students in this activity gained an appreciation of the importance of error identification in pharmacy practice. This is an example of how prejudgements were reformed in response to the IPE simulation. Previous literature has shown that attitudes towards hierarchies can improve after interprofessional sessions; however, one longitudinal study proved that changes in perception returned to negative after 4 months [8]. This highlights the effect established stereotypes among professions can have on newer generations of healthcare students and workers.

### Limitations

As the data relies on focus group discussion, there is a risk of being open to response bias; however, interprofessional and uniprofessional focus groups were included; therefore, the authors were convinced of the honesty of the views and opinions expressed by the students. Given the theoretical orientation in this study, generalisability was never an objective. Moreover, this study was exploratory in nature, illuminating medical and pharmacy students’ experiences of this simulation-based IPE activity. In so doing, we feel that our findings may trigger others to consider adapting or carrying out such a similar simulation in their own institution. Analysis was carried out by some members of the team who also created the simulation teaching innovation. Whilst this may introduce biases, this was minimised by reflexivity checks and that the evaluation was led by an individual who was not part of the team that created the simulation innovation.

### Future research

This interprofessional-simulated activity has been adopted by the School of Pharmacy and the School of Medicine and integrated into level 3 pharmacy and level 4 medical curricula. Future research will see the continuation of the activity for the next cohort of students, addressing feedback from the focus group data. A longitudinal study of an interprofessional group in their years of clinical practice could yield interesting results, answering the questions of longevity in the changes in attitude and values of professional roles seen in this study. Furthermore, exploring the impact of this simulation innovation on other healthcare professionals who prescribe (e.g. independent nurse prescribers) would be worthy. Whilst impact on prescribing errors was beyond the remit of this evaluation, this would be an important area to consider in future research, particularly given the notion that prescribing is embedded in social settings that make the performance of this apparently *simple* task, complex and prone to error [1].

## Conclusion

This innovative simulated IPE activity was designed for senior pharmacy and medical students and aimed to develop their consultation, prescription writing and dispensing skills in an environment that fostered collaboration and mentorship. Driven by healthcare training needs, this innovation broadens the scope of simulation-based learning, harnessing interprofessional education in the important topic of prescribing in the community. Both professions felt that the interprofessional activity helped to improve their own clinical skills and, in turn, learn to trust in the expertise of their colleagues. Having participated in the session, students expressed a wider knowledge of the roles their professions engage in. In addition, pharmacy and medical students alike believed the activity would contribute to strengthening their future cooperation together striving for the mutual goal of improved patient-centred care—the ‘person’ behind the prescription.

## Abbreviations

GP: General practitioner
IPE: Interprofessional education
QUB: Queen’s University Belfast
SBE: Simulation-based education
SP: Simulated patient

## References

[1] Dornan T, Ashcroft D, Heathfield H, et al. An in depth investigation into causes of prescribing errors by foundation trainees in relation to their medical education. http://www.gmc-uk.org/FINAL_Report_prevalence_and_causes_of_prescribing_errors.pdf_28935150.pdf. Accessed 4 Mar 2017.

[2] Reeves S, Perrier L, Goldman J, Freeth D, Zwarenstein M, Reeves S (2013). Interprofessional education: effects on professional practice and healthcare outcomes (update). Cochrane Database of Systematic Reviews.

[3] Endacott R, Scholes J, Buykx P, Cooper S, Kinsman L, McConnell-Henry T (2010). Final-year nursing students’ ability to assess, detect and act on clinical cues of deterioration in a simulated environment. J Adv Nurs.

[4] Wayne DB, Didwania A, Feinglass J, Fudala MJ, Barsuk JH, McGaghie WC (2008). Simulation-based education improves quality of care during cardiac arrest team responses at an academic teaching hospital. Chest.

[5] Rosenthal ME, Adachi M, Ribaudo V, Mueck JT, Schneider RF, Mayo PH (2006). Achieving house staff competence in emergency airway management using scenario based simulation training. Chest.

[6] Commission on future models of care delivered through pharmacy November 2013. https://www.rpharms.com/Portals/0/RPS%20document%20library/Open%20access/Publications/Now%20or%20Never%20-%20Report.pdf. Accessed 4 Mar 2017.

[7] Wellmon R, Gilin B, Knauss L, Inman LM. Changes in student attitudes toward interprofessional learning and collaboration arising from a case-based educational experience. J Allied Health. 2012;41(1):26–34. 22544405

[8] Reeves S, Fletcher S, Barr H (2016). A BEME systematic review of the effects of interprofessional education: BEME guide no. 39. Med Teach.

[9] Curran VR, Sharpe D, Flynn K, Button P (2010). A longitudinal study of the effect of an interprofessional education curriculum on student satisfaction and attitudes towards interprofessional teamwork and education. J Interprof Care.

[10] Honan L, Fahs DB, Talwalkar JS, Kayingo G (2015). Interprofessional learning: perceptions of first year health students. J Nurs Educ Pract.

[11] (Pro) RG, (Con) EP. Canadian Journal of Hospital Pharmacy. Vol 69. Canadian Society of Hospital Pharmacists; 2016. http://cjhp-online.ca/index.php/cjhp/article/view/1598/2441. Accessed 4 Mar 2017.

[12] Birley KJ, Moreiras J, Fertleman CR, Bates I (2014). Integrated pharmacy and medical student practical prescribing teaching. Med Educ.

[13] Rotz ME, Dueñas GG, Grover AB, Headly A, Parvanta CF (2015). Exploring first-year pharmacy and medical students’ experiences during a longitudinal interprofessional education program. Curr Pharm Teach Learn.

[14] Van Winkle LJ, Bjork BC, Chandar N (2012). Interprofessional workshop to improve mutual understanding between pharmacy and medical students. Am J Pharm Educ.

